# Updated measuring motor, sensory and sphincter dysfunctions in patients with cervical myelopathy using the modified Japanese Orthopedic Association (mJOA) score: a validation study

**Published:** 2012-11

**Authors:** Parisa Azimi, Sohrab Shahzadi, Edward C. Benzel, Ali Montazari

**Affiliations:** ^*a*^Department of Neurosurgery, Shahid Beheshti University of Medical Sciences, Tehran, Iran.; ^*b*^Cleveland Clinic Foundation, Department of Neurosurgery, Cleveland, Ohio,USA.; ^*c*^Mental Health Research Group, Health Metrics Research Centre, Iranian Institute for Health Sciences Research, ACECR, Tehran, Iran.

## Abstract

**Background:**

Cervical myelopathy population (CMP) is a common cause of significant clinical morbidity. This study aimed to translate and validate the modified Japanese Orthopedic Association score (mJOA) English version in Iran.

**Methods:**

This was a prospective clinical validation study. Psychometric properties of the questionnaire were assessed using internal consistency; inter-observer reliability, discriminant validity, and responsive ness of the questionnaire to change.

**Results:**

A total of 63 patients were studied. The mean mJOA score was 9.82 (SD = 1.0). Internal consistency of the mJOA as measured by Cronbach’s alpha coefficient was satisfactory (alpha = 0.77). Inter-observer agreement as measured by Kappa statistics also was found to be acceptable (0.75). Known-groups comparison was performed to test discriminant validity and the findings indicated that the mJOA was able to differentiate between males and females and different age groups as expected (P less than 0.0001). Finally the questionnaire was found to be responsive to change as patients’ pre- and post-operative scores on the mJOA (P less than 0.0001).

**Conclusions:**

The Iranian version of mJOA performed well and the findings suggest that it is a reliable and valid measure of motor, sensory and sphincter dysfunctions among CMP patients.

**Keywords:**

Cervical myelopathy patients, Grading scale, Modified japanese orthopedic Association score


					Volume 4, Suppl. 1 Nov 2012
Publisher: Kermanshah University of Medical Sciences
URL: http://www.jivresearch.org
Copyright:
Congress of Iranian Neurosurgeons, 14-16 November 2012, Kermanshah, Iran
Paper No. 41
Updated measuring motor, sensory and sphincter
dysfunctions in patients with cervical myelopathy using the
modified Japanese Orthopedic Association (mJOA) score: a
validation study
Parisa Azimi a,*
, Sohrab Shahzadi a
, Edward C. Benzel b
, Ali Montazari c
a
Department of Neurosurgery, Shahid Beheshti University of Medical Sciences, Tehran, Iran.
b
Cleveland Clinic Foundation, Department of Neurosurgery, Cleveland, Ohio,USA.
c
Mental Health Research Group, Health Metrics Research Centre, Iranian Institute for Health Sciences Research, ACECR,
Tehran, Iran.
Abstract:
Background: Cervical myelopathy population (CMP) is a common cause of significant clinical morbidity. This study
aimed to translate and validate the modified Japanese Orthopedic Association score (mJOA) English version in Iran.
Methods: This was a prospective clinical validation study. Psychometric properties of the questionnaire were assessed
using internal consistency; inter-observer reliability, discriminant validity, and responsive ness of the questionnaire to
change.
Results: A total of 63 patients were studied. The mean mJOA score was 9.82 (SD = 1.0). Internal consistency of the
mJOA as measured by Cronbach's alpha coefficient was satisfactory (alpha = 0.77). Inter-observer agreement as
measured by Kappa statistics also was found to be acceptable (0.75). Known-groups comparison was performed to
test discriminant validity and the findings indicated that the mJOA was able to differentiate between males and
females and different age groups as expected (P less than 0.0001). Finally the questionnaire was found to be
responsive to change as patients' pre- and post-operative scores on the mJOA (P less than 0.0001).
Conclusions: The Iranian version of mJOA performed well and the findings suggest that it is a reliable and valid
measure of motor, sensory and sphincter dysfunctions among CMP patients.
Keywords:
Cervical myelopathy patients, Grading scale, Modified japanese orthopedic Association score
*Corresponding Author at:
Parisa Azimi: Department of Neurosurgery, Imam-Hossain Hospital, Shahid-Beheshti University of Medical Sciences, Imam-Hossain sq., Tehran, Iran.
Email: Parisa.azimi@gmail.com, (Azimi P.).

				

